# Dynamic Characteristics of a Small-Size Beam Mounted on an Accelerating Structure

**DOI:** 10.3390/mi14040780

**Published:** 2023-03-30

**Authors:** Sajid Ali, Muhammad A. Hawwa

**Affiliations:** 1 Mechanical and Energy Engineering Department, Imam Abdulrahman Bin Faisal University, Dammam 31441, Saudi Arabia; 2 Department of Mechanical Engineering and IRC for Advanced Materials, King Fahd University of Petroleum & Minerals, Dhahran 31261, Saudi Arabia

**Keywords:** dynamic analysis, coordinate transformation, bifurcation, transverse response, moving support

## Abstract

This study focuses on the nonlinear vibration of a small-size beam hosted in a high-speed moving structure. The equation of the beam’s motion is derived using the coordinate transformation. The small-size effect is introduced by applying the modified coupled stress theory. The equation of motion involves quadratic and cubic terms due to mid-plane stretching. Discretization of the equation of motion is achieved via the Galerkin method. The impact of several parameters on the non-linear response of the beam is investigated. Bifurcation diagrams are used to investigate the stability of the response, whereas softening/hardening characteristics of the frequency curves are used as an indication of nonlinearity. Results indicate that increasing the magnitude of the applied force tends to signify the nonlinear hardening behavior. In terms of the periodicity of the response, at a lower amplitude of the applied force, the response appears to be a one-period stable oscillation. Increasing the length scale parameter, the response moves from chaotic to period-doubling to the stable one-period response. The impact of the axial acceleration of the moving structure on the stability as well as on the nonlinearity of the response of the beam is also investigated.

## 1. Introduction

Several small-size electro-mechanical devices embrace beam-shaped structures. Examples include sensors, actuators, resonators, accelerometers, gyroscopes, and energy harvesters. When these devices are hosted in high-speed vehicles, such as space shuttles, missiles, and hyper loops, their beam-shaped components are prone to changes in dynamic characteristics. The observation of these changes has an important value in deciding whether the small-size device is still performing accurately. A legitimate question that needs an answer, for example, is: would a cell phone accelerometer function appropriately if it is inside a high-speed vehicle? From a modeling perspective, the small-size beams have to be analyzed through a vibrating, while moving, structure. In a special case of interest, a beam that is aligned with the hosing vehicle can transversely vibrate while axially moving.

At a macro scale, numerous studies have been devoted to dynamic analyses of axially moving slender beams for cases including power transmitting belts, cutting saws, and roll-to-roll processing. Comprehensive reviews of the dynamics of axially moving structures have been presented in [1,2,3,4,5]. Ulsoy [6] investigated the effect of elastic coupling between two spans on the out-of-plane vibration of a moving beam. The stability characteristics of moving beam-like structures were investigated in [7,8,9] under various excitation frequencies. A moving beam with viscoelastic support was considered in [10], and the transverse response of the beam under various excitation was investigated. Similarly, Zhang et al. [11] explored the non-linear dynamics of beams under harmonic excitation. The effect of fractional order on the response curves was used to perform a parametric study. A novel solution scheme to solve the hyperbolic partial differential equation representing the equation of motion of a moving beam was developed in [12]. It was shown that the finite difference method could effectively and efficiently be used to study the dynamic response of moving beams. While applying the distribution of power law, dynamic analysis of edge cracks moving beam was presented in [13]. The findings demonstrate growth in the crack depth, and an increase in the speed of the beam results in a significant drop in the natural frequencies. The transverse response in terms of bifurcation and resonance for a moving ferromagnetic plate was presented by Cao and Hu [14]. The transverse response of a moving cantilever beam under the effect of distributed mass was numerically investigated in [15]. Through the analysis, the phenomena of energy separation were observed. It was observed that the elastic coupling strongly influences the dynamics response.

Axially moving elastic beams under various end conditions were investigated, and modal functions were developed to obtain the natural frequencies [16,17]. Axially moving nanobeams were investigated by Lim et al. [18]. An increase in natural frequencies and stiffness of the beam was observed with an increase in the nanoscale parameter. Li et al. [19] and Kiani [20] presented the modified nonlocal theory to investigate the dynamics of nanobeams. The free response of a small-size beam immersed in a denser fluid is studied in [21]. Analyzing the modes of vibration, it was concluded that the effect of fluid on the lower modes was more significant than the higher modes of vibration. The dynamics of axially moving materials in printing applications had also been studied extensively [22,23,24]. Nonhomogeneous boundary conditions were used in the investigation of the dynamic characteristics of a moving beam by Zhang et al. [25].

The vibration of piezoelectric nanobeams was considered by Li [26] using the nonlocal elastic theory. The application of modified coupled stress theory was presented by Marynowski [27] on the dynamic of the microscale panel. Similarly, the size dependency of a moving beam was studied in [28] using the modified coupled stress theory. Damghanian et al. [29], via the application of modified stress theory, investigated the static as well as the dynamic response of a beam element. Nazari et al. [30] presented a novel solution technique that was solely based on the shear-deformation theory to explore the lateral response of moving plates. Liu et al. [31] compared the dynamic instabilities of clamped and simply supported axially moving nanoplates. The impact of the time-dependent longitudinal velocity of nanobeams on the frequencies was studied by Rezaee et al. [32] and Liu et al. [33,34]. Dynamic responses of micro/nano moving beams were considered in several recent studies [35,36,37]. The impact of thermal conditions on the vibration and stability of a microbeam was thoroughly studied in [35]. A stability analysis of the response was performed through the bifurcation diagrams. The case of a thermoelastic nanobeam having constant axial tension under simply support boundary conditions was considered in [36]. The geometrical nonlinearity of a functionally graded moving nanobeam was estimated by Ji et al. [37]. As a result of raising the nonlocal parameter, wave frequency and propagation velocity were found to decrease.

On a different research front, the dynamics of vehicles carrying packages have been considered. Mathematical models were proposed in [38,39] to determine the critical speed of the vehicle on irregular road profiles. Lak et al. [40] studied the vibration of the vehicle as a function of road unevenness. Dynamic analysis of a vehicle was performed by Barbosa [41] using the spectral method. Gaith [42] and Ragulskis et al. [43] studied the vibration of a loaded moving container and determined the critical speed of the vehicle. The relation between the vehicle load and the contact point of the vehicle with the road was studied by [44,45].

A small-size beam mounted on a moving structure can be considered as a package of small size inside a moving vehicle. As one can see, the pre-cited literature was either devoted to studying the vibrations of vehicles or focused on investigating the vibrations of axially moving beams. No significant attention could be reported on the vibration of payloads carried by moving vehicles. To the best of the authors’ literature survey, nothing was found on the vibration of small-size beams in high-speed transportation systems. For this reason, this study is devoted to studying the forced vibrations of a small-size beam, which is hosted in an axially moving means of transportation. More specifically, the small-size beam is modeled by applying the coordinate transformation to account for the hosting structure movement. This paper is arranged as follows. In Section 2, the equation of nonlinear transverse motion is first derived in the absolute coordinate system. Then, the coordinate transformation between the two coordinate systems is made. The Galerkin method is then employed to discretize the partial differential equation of motion into a set of ordinary differential equations. In Section 3, phase portrait, Poincaré section, frequency response, and bifurcation diagrams are used to investigate the impacts of the magnitude of the applied force, axial speed, axial acceleration, and some beam parameters on its dynamical behavior.

## 2. Mathematical Formulation

**Figure 1:**
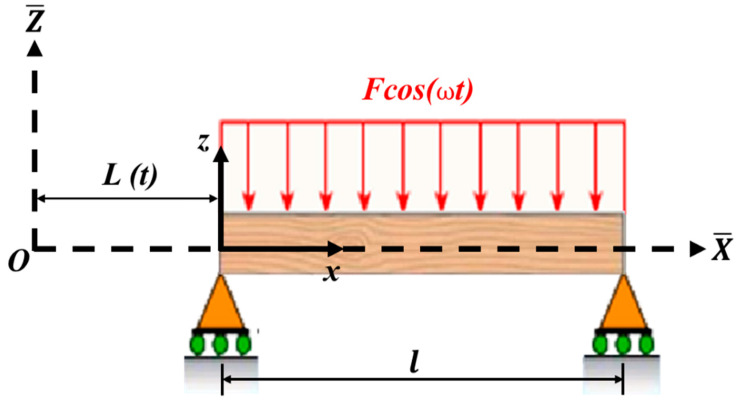
Small-size beam attached to a moving structure.

Figure 1 shows a small-size beam fixed in an axially moving structure. The length of the beam is *l*, flexural rigidity is *EI*, cross-sectional area is *A*, and density of the beam is ρ. The motion of the entire system (beam attached to the moving structure) is described by setting up two coordinate systems, the absolute coordinate system ($\overset{-}{X},\overset{-}{Z}$) and the moving coordinate system (*x*, *z*). The beam’s motion equation is first determined in absolute coordinates, following which the coordinate transformation between the coordinate systems is performed. Displacement of the beam in the lateral direction in the absolute coordinate system is w ($\overset{-}{X},\overset{-}{t}$).

If the small-size beam is loaded transversely with a harmonically varying force, $Fcos⍵\overset{-}{t},$ where F is the forcing function amplitude, and ⍵ is the frequency with which the force is applied. The beam is also loaded axially with a constant force, P. Then, the force and moment balances can be written as [1]:

(1) $$F_{\overset{-}{Z}}-\frac{\partial }{\partial \overset{-}{X}}\left(Q+P\frac{\partial w}{\partial \overset{-}{X}}\right)-Fcos⍵\overset{-}{t}=0$$

(2) $$Q=-EI\frac{\partial^{3}w}{\partial {\overset{-}{X}}^{3}}$$

Using (2) in (1),

(2a) $$F_{\overset{-}{Z}}-\frac{\partial }{\partial \overset{-}{X}}\left(-EI\frac{\partial^{3}w}{\partial {\overset{-}{X}}^{3}}+P\frac{\partial w}{\partial \overset{-}{X}}\right)-Fcos⍵\overset{-}{t}=0$$

Applying the total differential of time,

(3) $$v_{\overset{-}{Z}}=\frac{Dw}{D\overset{-}{t}}=\frac{\partial w}{\partial \overset{-}{t}}+\frac{\partial w}{\partial \overset{-}{X}}\frac{\partial \overset{-}{X}}{\partial \overset{-}{t}}=\frac{\partial w}{\partial \overset{-}{t}}+\dot{L}\frac{\partial w}{\partial \overset{-}{X}}$$

Now applying the material derivative,

(4a) $$a_{\overset{-}{Z}}=\frac{Dv_{\overset{-}{Z}}}{D\overset{-}{t}}=\frac{\partial v_{\overset{-}{Z}}}{\partial \overset{-}{t}}+\frac{\partial v_{\overset{-}{Z}}}{\partial \overset{-}{X}}\frac{\partial \overset{-}{X}}{\partial \overset{-}{t}}$$

(4b) $$a_{\overset{-}{Z}}=\frac{Dv_{\overset{-}{Z}}}{D\overset{-}{t}}=\frac{\partial }{\partial \overset{-}{t}}(\frac{\partial w}{\partial \overset{-}{t}}+\dot{L}\frac{\partial w}{\partial \overset{-}{X}})+\dot{L}\frac{\partial }{\partial \overset{-}{X}}(\frac{\partial w}{\partial \overset{-}{t}}+\dot{L}\frac{\partial w}{\partial \overset{-}{X}})$$

(4c) $$a_{\overset{-}{Z}}=\frac{Dv_{\overset{-}{Z}}}{D\overset{-}{t}}=\frac{\partial^{2}w}{\partial {\overset{-}{t}}^{2}}+\ddot{L}\frac{\partial w}{\partial \overset{-}{X}}+\dot{L}\frac{\partial^{2}w}{\partial \overset{-}{X}\partial \overset{-}{t}}+\dot{L}\frac{\partial^{2}w}{\partial \overset{-}{X}\partial \overset{-}{t}}+{\dot{L}}^{2}\frac{\partial^{2}w}{\partial {\overset{-}{X}}^{2}}$$

(4d) $$a_{\overset{-}{Z}}=\frac{Dv_{\overset{-}{Z}}}{D\overset{-}{t}}=\frac{\partial^{2}w}{\partial {\overset{-}{t}}^{2}}+2\dot{L}\frac{\partial^{2}w}{\partial \overset{-}{X}\partial \overset{-}{t}}+{\dot{L}}^{2}\frac{\partial^{2}w}{\partial {\overset{-}{X}}^{2}}+\ddot{L}\frac{\partial w}{\partial \overset{-}{X}}$$

(4) $$a_{\overset{-}{Z}}=({\left(\frac{\partial }{\partial \overset{-}{t}}+\dot{L}\frac{\partial }{\partial \overset{-}{X}}\right)}^{2}+\ddot{L}\frac{\partial }{\partial \overset{-}{X}})w$$

Using (4) in (2a),

(5a) $$\rho A\left(\left({\left(\frac{\partial }{\partial \overset{-}{t}}+\dot{L}\frac{\partial }{\partial \overset{-}{X}}\right)}^{2}+\ddot{L}\frac{\partial }{\partial \overset{-}{X}}\right)w\right)-\frac{\partial }{\partial \overset{-}{X}}\left(-EI\frac{\partial^{3}w}{\partial {\overset{-}{X}}^{3}}+P\frac{\partial w}{\partial \overset{-}{X}}\right)=Fcos⍵\overset{-}{t}$$

(5b) $$\rho A\left(\frac{\partial^{2}w}{\partial {\overset{-}{t}}^{2}}+2\dot{L}\frac{\partial^{2}w}{\partial \overset{-}{X}\partial \overset{-}{t}}+{\dot{L}}^{2}\frac{\partial^{2}w}{\partial {\overset{-}{X}}^{2}}+\ddot{L}\frac{\partial w}{\partial \overset{-}{X}}\right)+EI\frac{\partial^{4}w}{\partial {\overset{-}{X}}^{4}}-P\frac{\partial^{2}w}{\partial {\overset{-}{X}}^{2}}=Fcos⍵\overset{-}{t}$$

(5c) $$\rho A\frac{\partial^{2}w}{\partial {\overset{-}{t}}^{2}}+2\rho A\dot{L}\frac{\partial^{2}w}{\partial \overset{-}{X}\partial \overset{-}{t}}+\rho A{\dot{L}}^{2}\frac{\partial^{2}w}{\partial {\overset{-}{X}}^{2}}+\rho A\ddot{L}\frac{\partial w}{\partial \overset{-}{X}}+EI\frac{\partial^{4}w}{\partial {\overset{-}{X}}^{4}}-P\frac{\partial^{2}w}{\partial {\overset{-}{X}}^{2}}=Fcos⍵\overset{-}{t}$$

(5d) $$\rho A\frac{\partial^{2}w}{\partial {\overset{-}{t}}^{2}}+2\rho A\dot{L}\frac{\partial^{2}w}{\partial \overset{-}{X}\partial \overset{-}{t}}+(\rho A{\dot{L}}^{2}-P)\frac{\partial^{2}w}{\partial {\overset{-}{X}}^{2}}+\rho A\ddot{L}\frac{\partial w}{\partial \overset{-}{X}}+EI\frac{\partial^{4}w}{\partial {\overset{-}{X}}^{4}}=Fcos⍵\overset{-}{t}$$

The moveable coordinate system has been defined as

(5) $$\left\{\begin{array}{l} x=\overset{-}{X}-L(t)\\ z=\overset{-}{Z}\\ t=\overset{-}{t} \end{array}\right.$$

This leads to defining derivatives in the following forms:

(6) $$\frac{\partial }{\partial \overset{-}{X}}=\frac{\partial }{\partial x};\frac{\partial }{\partial \overset{-}{t}}=\frac{\partial }{\partial t}+\frac{\partial }{\partial x}\frac{\partial x}{\partial t}=\frac{\partial }{\partial t}-\dot{L}\frac{\partial }{\partial x}$$

Substituting (7) in (5d),

(7) $$\rho A{\left[\frac{\partial }{\partial t}-\dot{L}\frac{\partial }{\partial x}\right]}^{2}w+2\rho A\dot{L}\frac{\partial }{\partial x} [\frac{\partial }{\partial t}-\dot{L}\frac{\partial }{\partial x}]w+\left({\rho A\dot{L}}^{2}-P\right)\frac{\partial^{2}w}{\partial x^{2}}\;+\rho A\ddot{L}\frac{\partial w}{\partial x}+EI\frac{\partial^{4}w}{\partial x^{4}}=Fcos⍵t$$

Recognizing $\dot{L}$ as v and $\ddot{L}$ as ***a*** (i.e., axial speed and acceleration of moving coordinate with respect to absolute coordinate), (8) becomes
$\rho A\frac{\partial^{2}w}{\partial t^{2}}-2\rho Av\frac{\partial^{2}w}{\partial x\partial t}+{\rho Av}^{2}\frac{\partial^{2}w}{\partial x^{2}}+2\rho Av\frac{\partial^{2}w}{\partial x\partial t}-2{\rho Av}^{2}\frac{\partial^{2}w}{\partial x^{2}}$(8)      
$+{\rho Av}^{2}\frac{\partial^{2}w}{\partial x^{2}}-P\frac{\partial^{2}w}{\partial x^{2}}+\rho Aa\frac{\partial w}{\partial x}+EI\frac{\partial^{4}w}{\partial x^{4}}=Fcos⍵t$


Considering mid-plane stretching effect, (9) can be written in the following simplified form:

(9) $$\rho A\frac{\partial^{2}w}{\partial t^{2}}+\rho Aa\frac{\partial w}{\partial x}-P\frac{\partial^{2}w}{\partial x^{2}}+EI\frac{\partial^{4}w}{\partial x^{4}}=Fcos⍵t$$

Now, modified couple stress theory [2] is applied to incorporate the small-size effect, and the equation for the transverse motion becomes

(10) $$\left(AG{\ell_{s}}^{2}+EI\right)w_{xxxx}+\rho Aw_{tt}+\rho Aaw_{x}-Pw_{xx}\;-AEw_{xx}\int_{0}^{L}\frac{1}{2}w_{x}^{2}dx=Fcos⍵t$$

Equation (11) defines the equation of motion of micro/nano beam attached to a moving structure. Normalization of Equation (11) is performed by introducing the following variables:

(11) $$\hat{w}=\frac{w}{l},\;\hat{x}=\frac{x}{l},\;\hat{t}=\frac{t}{T}.$$

Now substituting Equation (12) into Equation (11) and utilizing the parameters given in Table 1,

(12) $$\begin{array}{c} \left(\zeta +1\right){\hat{w}}_{\hat{x}\hat{x}\hat{x}\hat{x}}+{\hat{w}}_{\hat{t}\hat{t}}+\hat{a}{\hat{w}}_{\hat{x}}-\hat{P}{\hat{w}}_{\hat{x}\hat{x}}-\alpha {\hat{w}}_{\hat{x}\hat{x}}\int_{0}^{1}{\hat{w}}_{\hat{x}}^{2}dx\\ =\hat{F}cos\Omega t \end{array}$$

Equation (13) is further simplified by ignoring the hat notation:

(13) $$\begin{array}{c} \left(\zeta +1\right)w_{xxxx}+w_{tt}+aw_{x}-Pw_{xx}-\alpha w_{xx}\int_{0}^{1}w_{x}^{2}dx\\ =Fcos\Omega t \end{array}$$

By applying the Galerkin method, Equation (14) was discretized into a system of ordinary differential equations. In Galerikin method, first, the transverse response of the beam is given as

(14) $$w\left(x,t\right)=\sum_{i=1}^{n}q_{i}\left(t\right)\phi_{i}\left(x\right)$$

where $q_{i}\left(t\right)$ represents the non-dimensional modal coordinates. Mode shapes of the displacement response are given as

(15) $$\phi_{i}\left(x\right)=\sin\left(i\pi x\right)$$

Substituting Equation (16) into Equation (15),

(16) $$\int_{0}^{1}\phi_{j}\left(\zeta +1\right)\sum_{i=1}^{n}q_{i}\phi_{i}^{’’’’}dx+\int_{0}^{1}\phi_{j}\sum_{i=1}^{n}{\ddot{q}}_{i}\phi_{i}dx+a\int_{0}^{1}\phi_{j}\sum_{i=1}^{n}q_{i}\phi_{i}^{’}dx\;-P\int_{0}^{1}\phi_{j}\sum_{i=1}^{n}q_{i}\phi_{i}^{’’}dx+\alpha \int_{0}^{1}\phi_{j} [\sum_{i=1}^{n}q_{i}\phi_{i}^{’’}\int_{0}^{1}{\left(\sum_{i=1}^{n}q_{i}\phi_{i}^{’}\right)}^{2}dx]dx\;\begin{array}{c} =F\int_{0}^{1}\phi_{j}cos\Omega tdx. \end{array}$$

Using 4-term expansion (*n* = 4), Equation (17) becomes
$\left(\zeta +1\right)\int_{0}^{1}\sin\left(\pi x\right)[q_{1}\pi^{4}\sin\left(\pi x\right)$(17)      
$+q_{2}16\pi^{4}\sin\left(2\pi x\right)+q_{3}81\pi^{4}\sin\left(3\pi x\right)$
      
$+q_{4}256\pi^{4}sin(4\pi x)]dx$
      
$+\int_{0}^{1}\sin\left(\pi x\right)[{\ddot{q}}_{1}\pi \sin\left(\pi x\right)$
      
$+{\ddot{q}}_{2}\sin\left(2\pi x\right)+{\ddot{q}}_{3}\sin\left(3\pi x\right)+{\ddot{q}}_{4}\sin\left(4\pi x\right)]dx$
      
$+a\int_{0}^{1}\sin\left(\pi x\right)[q_{1}\pi \cos\left(\pi x\right)$
      
$+q_{2}2\pi \cos\left(2\pi x\right)+q_{3}3\pi cos(3\pi x)$
      
${+q}_{4}4\pi cos(4\pi x)]dx$
      
$-P\int_{0}^{1}\sin\left(\pi x\right)[q_{1}(-{9\pi }^{2})\sin\left(\pi x\right)$
      
$+q_{2}(-4\pi^{2})\sin\left(2\pi x\right)+q_{3}(-{9\pi }^{2})\sin\left(3\pi x\right)$
      
$+q_{4}({-16\pi }^{2})sin(4\pi x)]dx$
      
$-\alpha \int_{0}^{1}\sin\left(\pi x\right)[[q_{1}(-\pi^{2})\sin\left(\pi x\right)$
      
$+q_{2}(-4\pi^{2})\sin\left(2\pi x\right)$
      
$+q_{3}(-{9\pi }^{2})\sin\left(3\pi x\right)$
      
$+q_{4}(-{16\pi }^{2})\sin\left(4\pi x\right)]\int_{0}^{1}[q_{1}\pi \cos\left(\pi x\right)$
      
$+q_{2}2\pi \cos\left(2\pi x\right)$
      
$+q_{3}3\pi \cos\left(3\pi x\right)+q_{4}4\pi \cos\left(4\pi x\right){]}^{2}dx]dx$
      
$=Fcos\Omega t\int_{0}^{1}\sin\left(\pi x\right)dx$
$\left(\zeta +1\right)\int_{0}^{1}\sin\left(2\pi x\right)[q_{1}\pi^{4}\sin\left(\pi x\right)$(18)      
$+q_{2}16\pi^{4}\sin\left(2\pi x\right)+q_{3}81\pi^{4}\sin\left(3\pi x\right)$
      
${+q}_{4}256\pi^{4}sin(4\pi x)]dx$
      
$+\int_{0}^{1}\sin\left(2\pi x\right)[{\ddot{q}}_{1}\pi \sin\left(\pi x\right)$
      
$+{\ddot{q}}_{2}\sin\left(2\pi x\right)+{\ddot{q}}_{3}\sin\left(3\pi x\right)+{\ddot{q}}_{4}\sin\left(4\pi x\right)]dx$
      
$+a\int_{0}^{1}\sin\left(2\pi x\right)[q_{1}\pi \cos\left(\pi x\right)$
      
$+q_{2}2\pi \cos\left(2\pi x\right)+q_{3}3\pi cos(3\pi x)$
      
$+q_{4}4\pi cos(4\pi x)]dx$
      
$-P\int_{0}^{1}\sin\left(2\pi x\right)[q_{1}(-\pi^{2})\sin\left(\pi x\right)$
      
$+q_{2}(-4\pi^{2})\sin\left(2\pi x\right)+q_{3}(-{9\pi }^{2})\sin\left(3\pi x\right)$
      
$+q_{4}({-16\pi }^{2})sin(4\pi x)]dx$
      
$-\alpha \int_{0}^{1}\sin\left(2\pi x\right)[[q_{1}(-\pi^{2})\sin\left(\pi x\right)$
      
$+q_{2}(-4\pi^{2})\sin\left(2\pi x\right)$
      
$+q_{3}(-{9\pi }^{2})\sin\left(3\pi x\right)$
      
$+q_{4}(-{16\pi }^{2})\sin\left(4\pi x\right)]\int_{0}^{1}[q_{1}\pi \cos\left(\pi x\right)$
      
$+q_{2}2\pi \cos\left(2\pi x\right)$
      
$+q_{3}3\pi \cos\left(3\pi x\right)+q_{4}4\pi \cos\left(4\pi x\right){]}^{2}dx]dx$
      
$=Fcos\Omega t\int_{0}^{1}\sin\left(2\pi x\right)dx$
$\left(\zeta +1\right)\int_{0}^{1}\sin\left(3\pi x\right)[q_{1}\pi^{4}\sin\left(\pi x\right)$(19)      
$+q_{2}16\pi^{4}\sin\left(2\pi x\right)+q_{3}81\pi^{4}\sin\left(3\pi x\right)$
      
${+q}_{4}256\pi^{4}sin(4\pi x)]dx$
      
$+\int_{0}^{1}\sin\left(3\pi x\right)[{\ddot{q}}_{1}\pi \sin\left(\pi x\right)$
      
$+{\ddot{q}}_{2}\sin\left(2\pi x\right)+{\ddot{q}}_{3}\sin\left(3\pi x\right)+{\ddot{q}}_{4}\sin\left(4\pi x\right)]dx$
      
$+a\int_{0}^{1}\sin\left(3\pi x\right)[q_{1}\pi \cos\left(\pi x\right)$
      
$+q_{2}2\pi \cos\left(2\pi x\right)+q_{3}3\pi cos(3\pi x)$
      
$+q_{4}4\pi cos(4\pi x)]dx$
      
$-P\int_{0}^{1}\sin\left(3\pi x\right)[q_{1}(-\pi^{2})\sin\left(\pi x\right)$
      
$+q_{2}(-4\pi^{2})\sin\left(2\pi x\right)+q_{3}(-{9\pi }^{2})\sin\left(3\pi x\right)$
      
$+q_{4}({-16\pi }^{2})sin(4\pi x)]dx$
      
$-\alpha \int_{0}^{1}\sin\left(3\pi x\right)[[q_{1}(-\pi^{2})\sin\left(\pi x\right)$
      
$+q_{2}(-4\pi^{2})\sin\left(2\pi x\right)$
      
$+q_{3}(-{9\pi }^{2})\sin\left(3\pi x\right)$
      
$+q_{4}(-{16\pi }^{2})\sin\left(4\pi x\right)]\int_{0}^{1}[q_{1}\pi \cos\left(\pi x\right)$
      
$+q_{2}2\pi \cos\left(2\pi x\right)$
      
$+q_{3}3\pi \cos\left(3\pi x\right)+q_{4}4\pi \cos\left(4\pi x\right){]}^{2}dx]dx$
      
$=Fcos\Omega t\int_{0}^{1}\sin\left(3\pi x\right)dx$
$\left(\zeta +1\right)\int_{0}^{1}\sin\left(4\pi x\right)[q_{1}\pi^{4}\sin\left(\pi x\right)$(20)      
$+q_{2}16\pi^{4}\sin\left(2\pi x\right)+q_{3}81\pi^{4}\sin\left(3\pi x\right)$
      
${+q}_{4}256\pi^{4}sin(4\pi x)]dx$
      
$+\int_{0}^{1}\sin\left(4\pi x\right)[{\ddot{q}}_{1}\pi \sin\left(\pi x\right)$
      
$+{\ddot{q}}_{2}\sin\left(2\pi x\right)+{\ddot{q}}_{3}\sin\left(3\pi x\right)+{\ddot{q}}_{4}\sin\left(4\pi x\right)]dx$
      
$+a\int_{0}^{1}\sin\left(4\pi x\right)[q_{1}\pi \cos\left(\pi x\right)$
      
$+q_{2}2\pi \cos\left(2\pi x\right)+q_{3}3\pi cos(3\pi x)$
      
$+q_{4}4\pi cos(4\pi x)]dx$
      
$-P\int_{0}^{1}\sin\left(4\pi x\right)[q_{1}(-\pi^{2})\sin\left(\pi x\right)$
      
$+q_{2}(-4\pi^{2})\sin\left(2\pi x\right)+q_{3}(-{9\pi }^{2})\sin\left(3\pi x\right)$
      
$+q_{4}({-16\pi }^{2})sin(4\pi x)]dx$
      
$-\alpha \int_{0}^{1}\sin\left(4\pi x\right)[[q_{1}(-\pi^{2})\sin\left(\pi x\right)$
      
$+q_{2}(-4\pi^{2})\sin\left(2\pi x\right)$
      
$+q_{3}(-{9\pi }^{2})\sin\left(3\pi x\right)$
      
$+q_{4}(-{16\pi }^{2})\sin\left(4\pi x\right)]\int_{0}^{1}[q_{1}\pi \cos\left(\pi x\right)$
      
$+q_{2}2\pi \cos\left(2\pi x\right)$
      
$+q_{3}3\pi \cos\left(3\pi x\right)+q_{4}4\pi \cos\left(4\pi x\right){]}^{2}dx]dx$
      
$=Fcos\Omega t\int_{0}^{1}\sin\left(4\pi x\right)dx$


Now, solving the individual terms in Equations (18)–(21), results in
${\ddot{q}}_{1}+\left[\left(\zeta +1\right)\pi^{4}+\pi^{2}P\right]q_{1}+\left[\frac{{\alpha \pi }^{4}}{2}\right]{q_{1}}^{3}+\left[{2\alpha \pi }^{4}\right]q_{1}{q_{2}}^{2}$(21)      
$+\left[\frac{{9\alpha \pi }^{4}}{2}\right]q_{1}{q_{3}}^{2}+\left[{8\alpha \pi }^{4}\right]{{q_{1}q}_{4}}^{2}-\left[\frac{8a}{3}\right]q_{2}$
      
$-\left[\frac{16a}{15}\right]q_{4}=\frac{4F}{\pi }cos\Omega t$
${\ddot{q}}_{2}+\left[\left(\zeta +1\right)16\pi^{4}+{4\pi }^{2}P\right]q_{2}+\left[{8\alpha \pi }^{4}\right]{q_{2}}^{3}+\left[{2\alpha \pi }^{4}\right]q_{2}{q_{1}}^{2}$(22)      
$+\left[{18\alpha \pi }^{4}\right]q_{2}{q_{3}}^{2}+\left[{32\alpha \pi }^{4}\right]{{q_{2}q}_{4}}^{2}+\left[\frac{4a}{15}*q_{1}\right]$
      
$-\left[\frac{12a}{5}*q_{3}\right]=0$
${\ddot{q}}_{3}+\left[\left(\zeta +1\right)81\pi^{4}+{9\pi }^{2}P\right]q_{3}+\left[\frac{{81\pi }^{4}}{2}\right]{q_{3}}^{3}+\left[\frac{{9\pi }^{4}}{2}\right]q_{3}{q_{1}}^{2}$(23)      
$+\left[{18\alpha \pi }^{4}\right]q_{3}{q_{2}}^{2}+\left[{72\alpha \pi }^{4}\right]{{q_{3}q}_{4}}^{2}+\left[\frac{24a}{5}*q_{2}\right]$
      
$-\left[\frac{48a}{7}*q_{4}\right]=\frac{4F}{3\pi }cos\Omega t$
${\ddot{q}}_{4}+\left[\left(\zeta +1\right)256\pi^{4}+{16\pi }^{2}P\right]q_{2}+\left[12{8\alpha \pi }^{4}\right]{q_{4}}^{3}+\left[{8\alpha \pi }^{4}\right]q_{4}{q_{1}}^{2}$(24)      
$+\left[{32\alpha \pi }^{4}\right]q_{4}{q_{2}}^{2}+\left[{72\alpha \pi }^{4}\right]{{q_{4}q}_{3}}^{2}+\left[\frac{16a}{15}*q_{1}\right]$
      
$+\left[\frac{16a}{7}*q_{3}\right]=0$


Second-order nonlinear differential equations of motion are represented by Equation (22) through (25). The beam’s nth mode of vibration in the transverse direction has the following fundamental natural frequency (ω):

(25) $$\omega_{n}=\sqrt{\left(\left(1+\zeta \right)*n^{4}\pi^{4})+{(n^{2}\pi }^{2}P\right)}$$

Regarding the reliability and verification of the results, study by An and Su [46] is considered. Equation (14) represents a partial differential equation of motion, involving quadratic nonlinearities. Compared with the equation of motion developed in [9], it is evident [46] that eliminating the small-size effect from the model developed in this study, the works in [46] can be considered as a special case of this study’s model. 

## 3. Results and Discussion

Dynamic characteristics of the small-size beam in terms of frequency response, Poincaré section, phase portrait, and bifurcation are investigated in this section by solving the system of Equations (22) and (23). The small-size beam characteristics are studied while varying the loading on the beam. Then, they are studied for different length scale parameters. Additionally, they are studied for different axial acceleration values. Typical nonlinear measures are used to understand the axially moving small-size beam, including frequency responses, phase portraits, Poincaré sections, and bifurcation diagrams. The range of parameter values employed in the numerical simulations is given in Table 2.

### 3.1. Varying the Magnitude of Applied Force (F)

**Figure 2:**
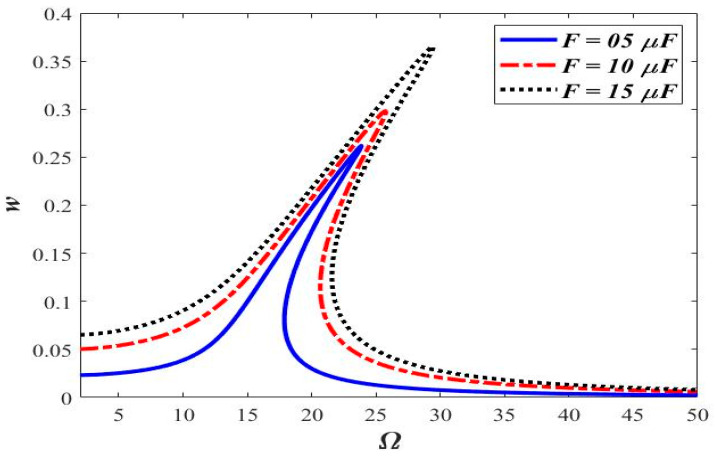
Comparison of frequency responses at different “*F*”, (*a* = 0.3 and $\ell_{s}=0.5\;h$.

Figure 2 shows the frequency-response comparison of the various magnitudes of applied force at axial acceleration “*a* = 0.3” and small-scale parameter ${“\ell }_{s}=0.5\;h”$. The frequency response of the beam with different forcing magnitudes is shown in Figure 2. Non-linearity in the form of the hardening behavior in the frequency response is observed.

A nonlinear hardening behavior is observed in the frequency response. Midplane stretching is the main contributing factor in the observed hardening frequency-response behavior. Increasing the magnitude of the applied force (*F* = 5 μN, 10 μN, and 15 μN) tends to signify the nonlinear hardening behavior. Furthermore, it is also observed that the maximum amplitude occurs at Ω = 15.6.

**Figure 3:**
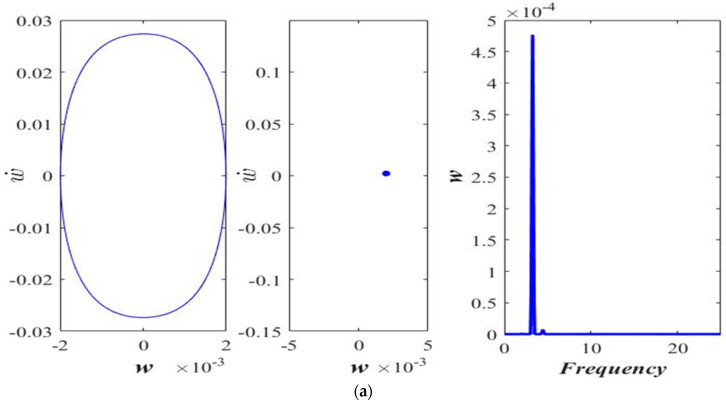

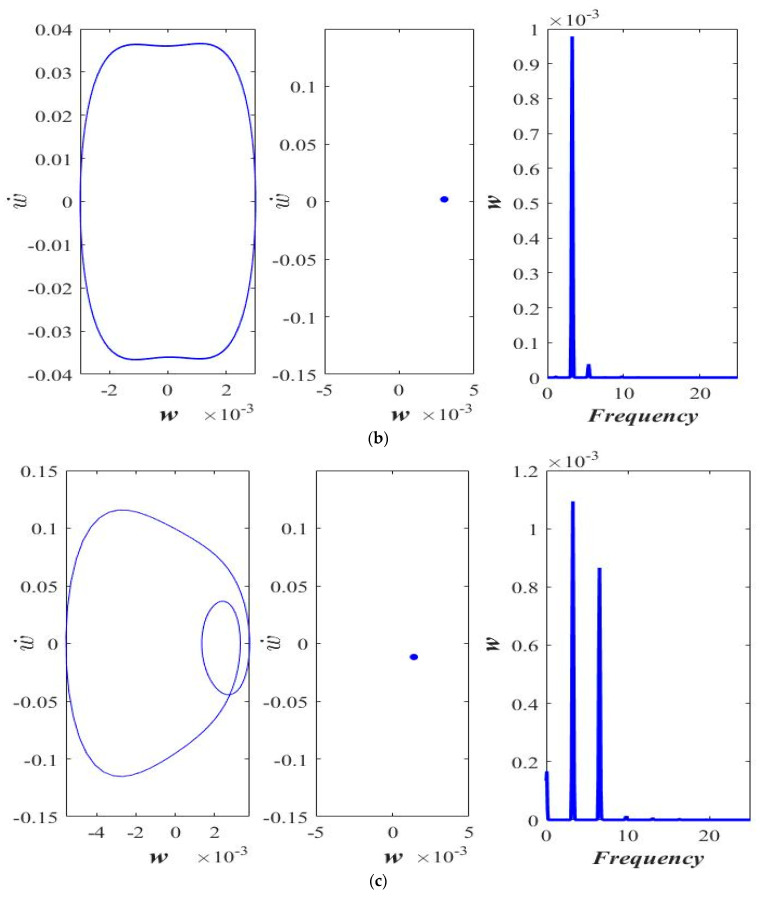

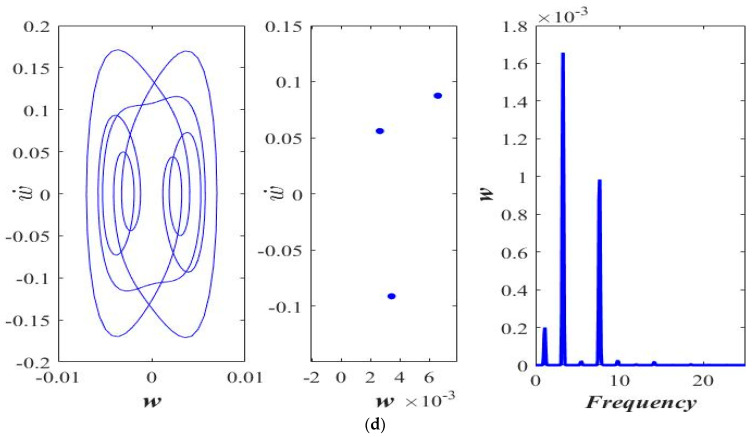
Phase portrait, Poincaré section, and frequency response at “*a* = 0.3” and “$\ell_{s}=0.5\;h$”, (**a**) *F* = 05 μN, (**b**) *F* = 15 μN, (**c**) *F* = 35 μN, and (**d**) *F* = 65 μN.

The effect of applied force magnitude on the response periodicity is evaluated via phase portrait and Poincaré map. The phase portrait (left), the Poincaré map (middle), and the frequency response (right) of the transverse motion are shown in Figure 3. At a lower amplitude of the applied force, the response appears to be periodic (Figure 3a,b). As the magnitude of the excitation force rises, the response shifts from periodic to period-doubling (Figure 3c,d). Although the periodicity of the response is disturbed by increasing the forcing amplitude, the response still seems to be far away from the chaos.

**Figure 4:**
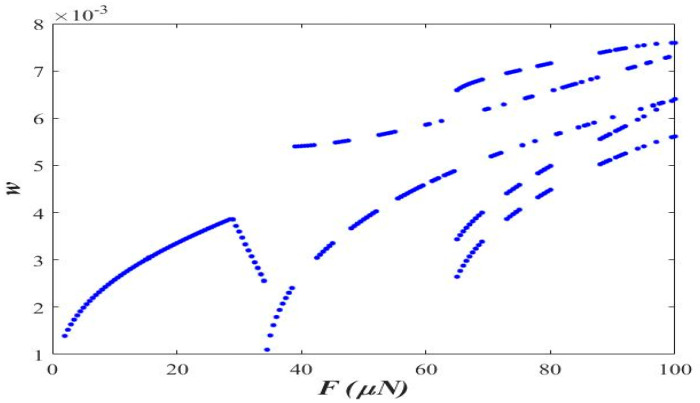
Bifurcation diagram (*a* = 0.3 and $\ell_{s}=0.5\;h$).

Figure 4 shows the bifurcation diagram in the applied force amplitude range of 0 to 100 μN at axial acceleration “*a* = 0.3” and small-scale parameter “$\ell_{s}=0.5\;h$”.

The observations made in Figure 3 are further supplemented with the help of the bifurcation diagram in the figure. As observed in Figure 4, the stable one-period and period-double are observed with a surge in the amplitude of the applied force. The period-doubling becomes more dominating when the applied force is above 100 μN. 

### 3.2. Varying Length Scale Parameter ($\ell_{s}$)

**Figure 5:**
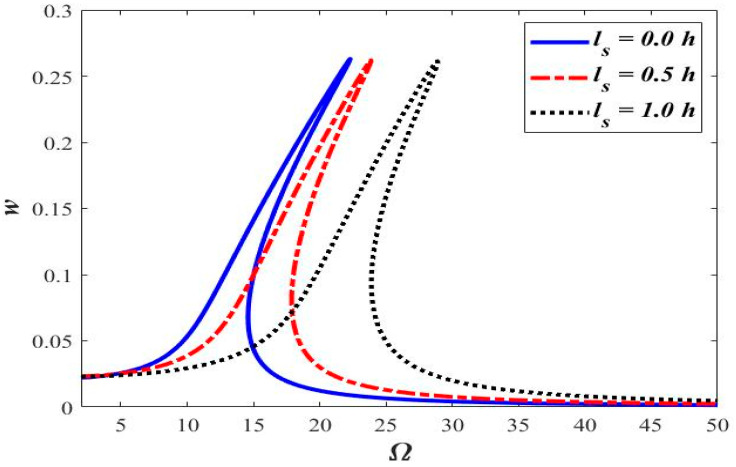
Frequency response curves at different “$\ell_{s}$”.

**Figure 6:**
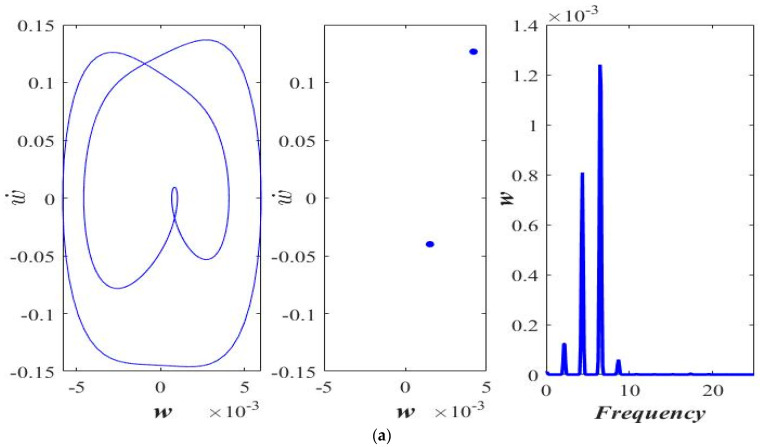

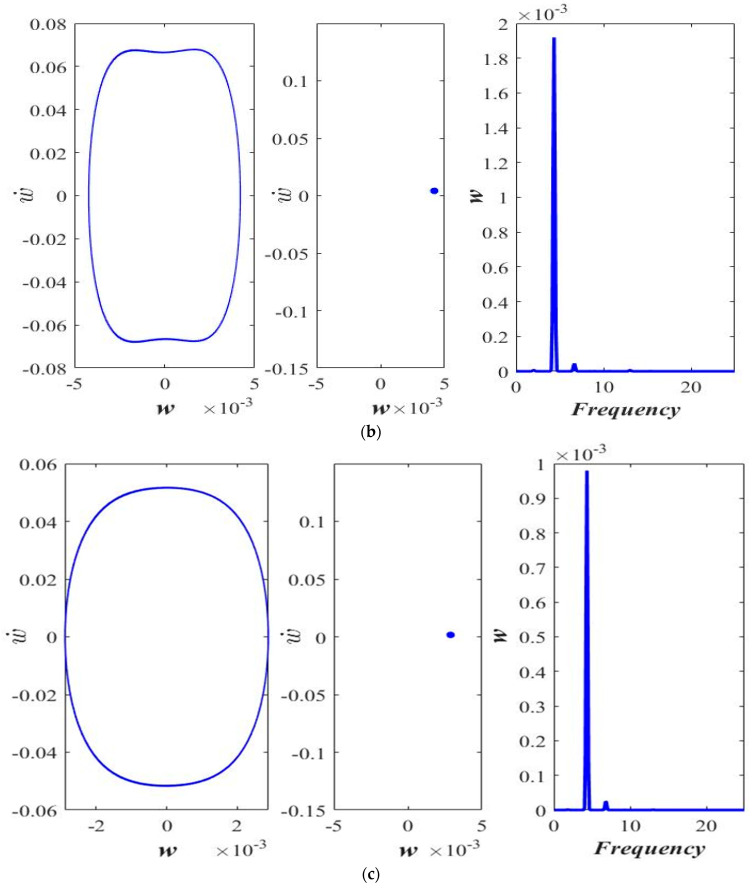
Phase portrait, Poincaré section, and frequency response at *a* = 0.3 and *F* = 25 μN, (**a**) $\ell_{s}=0.0,$ (**b**) $\ell_{s}=0.5\;h$, and (**c**) $\ell_{s}=1.0\;h$.

**Figure 7:**
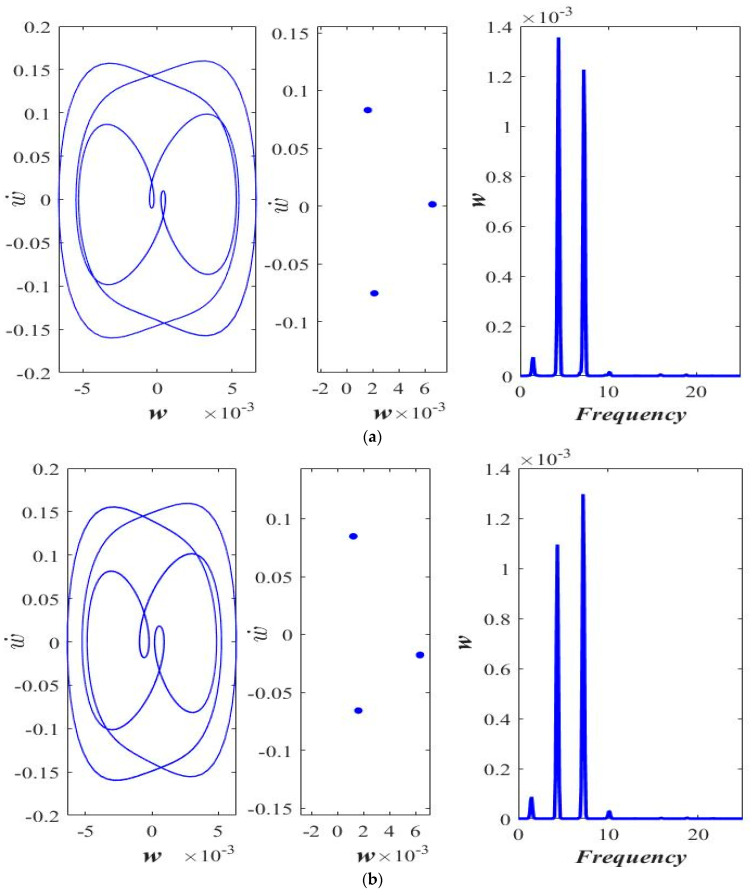

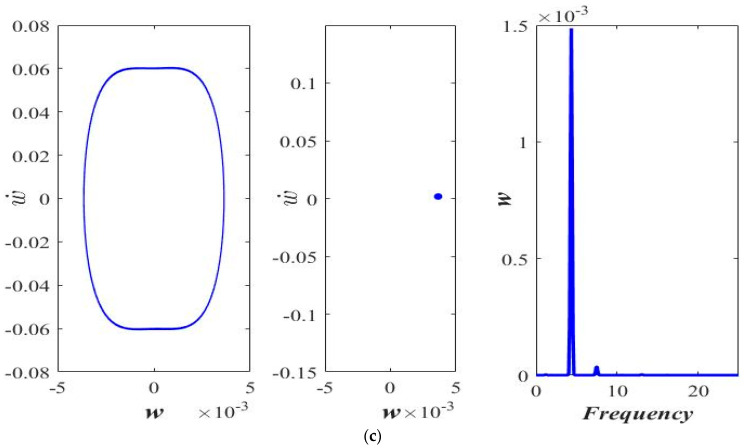
Phase portrait, Poincaré section, and frequency response at *a* = 0.3 and *F* = 40 μN, (**a**) $\ell_{s}=0.0,$ (**b**) $\ell_{s}=0.5\;h$, and (**c**) $\ell_{s}=1.0\;h$.

**Figure 8:**
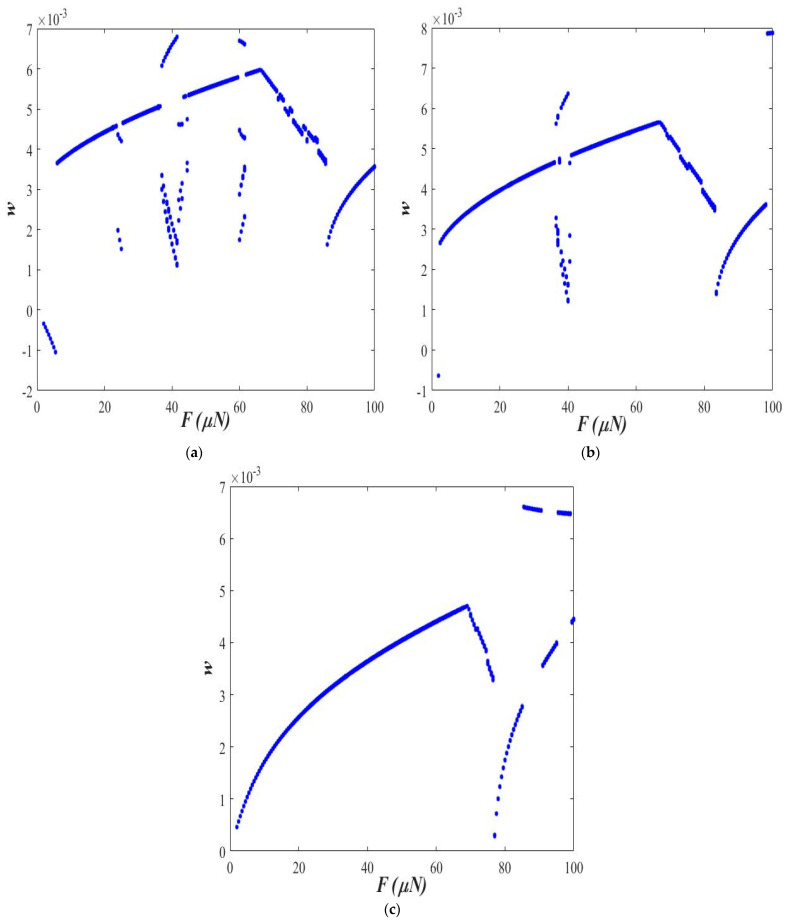
Bifurcation diagram at (**a**) $\ell_{s}=0.0$, (**b**) $\ell_{s}=0.5\;h$, and (**c**) $\ell_{s}=1.0\;h$ (*a* = 0.3).

The effect of the length-scale parameter ($\ell_{s}$) is presented in Figure 5, Figure 6, Figure 7 and Figure 8. Figure 5 shows the frequency-response comparison at $\ell_{s}=0.0,0.5\;h,and\;1.0\;h$.

Increasing the length-scale parameter results in a higher value of the excitation frequency corresponding to the maximum amplitude. Maximum amplitude occurs at Ω = 12.5, 15.6, and 21.8, corresponding to $\ell_{s}=0.0,0.5\;h,and\;1.0\;h,$ respectively. 

The impact of the $\ell_{s}$ is shown in Figure 6 and Figure 7 at lower (*F* = 25 μN) and higher (*F* = 40 μN) excitation force amplitude $F=Pcos(0.45t^{*})$.

The phase portrait (left), the Poincaré map (middle), and the frequency response (right) of the transverse motion are shown in Figure 6 and Figure 7. Interestingly, by increasing the $\ell_{s}$, the response of the axially moving microbeam moves from period-doublingthe response of the axially moving microbeam moves from period-doubling (Figure 6a) to a stable one-period response (Figure 6b,c).

At a higher amplitude of the applied force (*F* = 40 μN), the response is observed to be chaotic at $\ell_{s}=0.0$ (Figure 7a). With an increase in the value of $\ell_{s}$, the response of the beam first becomes period-doubling (Figure 7b), followed by stable 1-period (Figure 7c) corresponding to $\ell_{s}=0.5\;h$ and $\ell_{s}=1.0\;h$, respectively. Figure 8 shows the bifurcation diagram in the applied force amplitude range of 0 to 100 μN at axial acceleration “a = 0.3” and at small-scale parameters, $\ell_{s}=0.0$, Figure 8a, $\ell_{s}=0.5\;h$, Figure 8b, and $\ell_{s}=1.0\;h$, Figure 8c.

Observations made in Figure 7 can be further verified through the bifurcation diagrams shown in Figure 8. In the case of $\ell_{s}=0.0$, period-doubling bifurcation, quasi-periodic response, and chaotic behavior are observed by varying the amplitude of excitation force between 0 to 100 μN (Figure 8a). At $\ell_{s}=0.5*h$, a stable 1-period response in the lower (less than 40 μN) region of the excitation force amplitude, period-doubling response corresponding to the excitation force amplitude of 40 μN to 45 μN, and stable 1-period response for higher excitation force amplitude are observed, as shown in Figure 8b. By increasing the length-scale parameter further ($\ell_{s}=1.0*h$), the response of the beam becomes stable 1-period (Figure 8c).

### 3.3. Varying Acceleration

**Figure 9:**
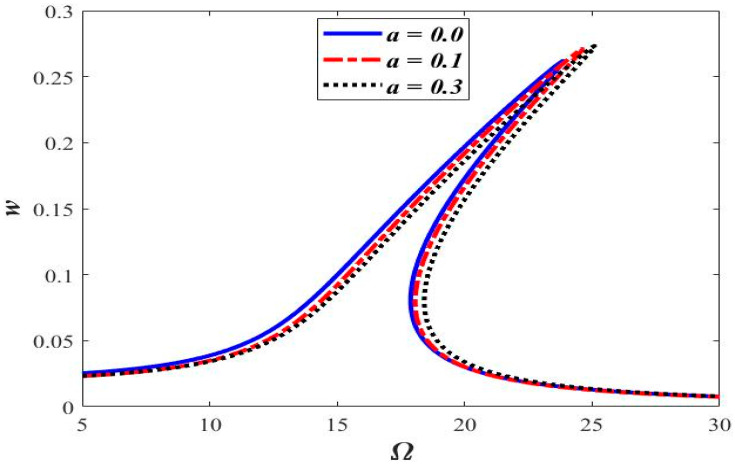
Frequency response curves at different acceleration “a”.

The frequency response of the beam with different axial accelerations is shown in Figure 9. Non-linearity in the form of the hardening behavior in the frequency response is observed to increase with increasing the magnitude of axial acceleration, i.e., the frequency response curve tends to move towards the right for higher values of axial acceleration, as shown in Figure 9.

**Figure 10:**
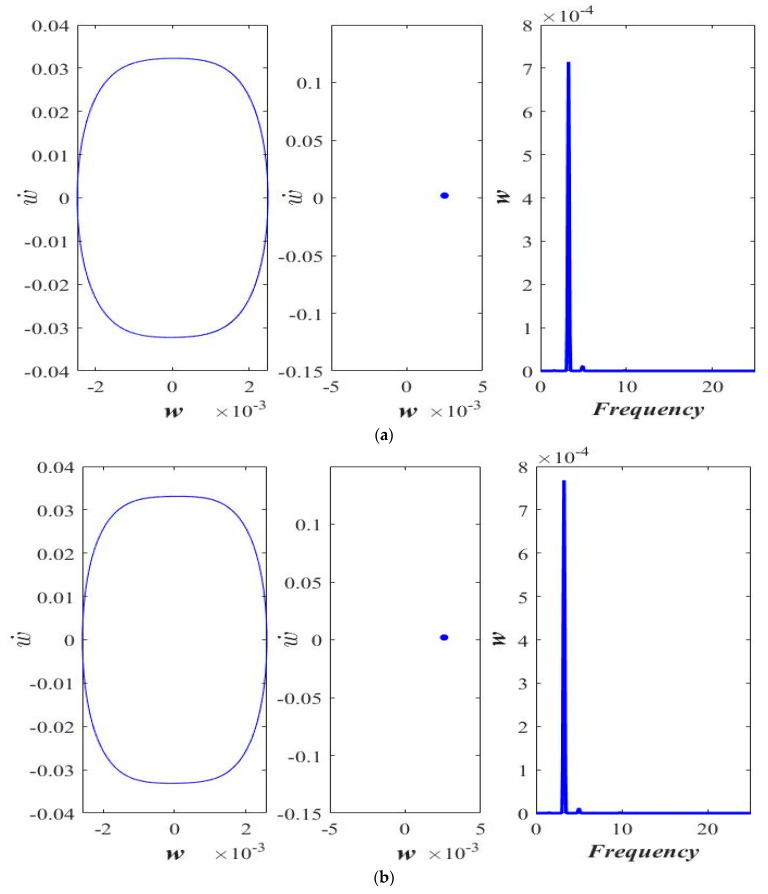

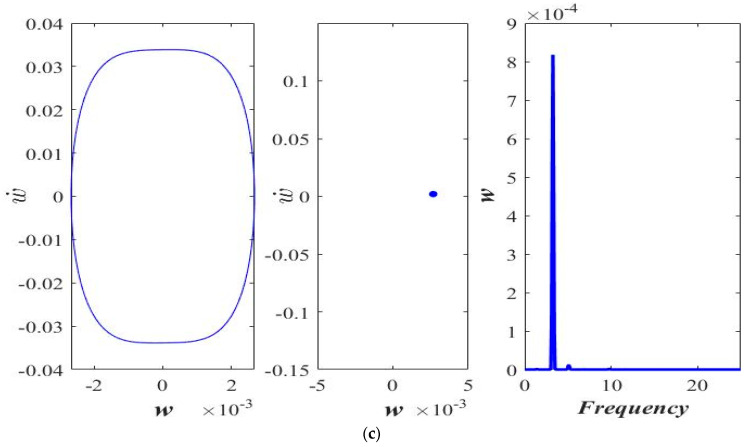
Phase portrait, Poincaré section, and frequency response at $\ell_{s}=0.5\;h$ and *F* = 10 μN, (**a**) *a* = 0.0, (**b**) *a* = 0.1, and (**c**) *a* = 0.3.

Figure 10 shows the phase portrait, the Poincaré map, and the frequency response of the transverse motion having different accelerations. Although the increase in acceleration tends to increase the hardening behavior (Figure 9), the periodicity of the response does not change with the increasing acceleration (Figure 10). A stable 1-period response is observed in the corresponding accelerations of *a* = 0.0, 0.1, and 0.3.

## 4. Conclusions

The transverse vibration of a small-size beam mounted on a moving structure is studied. The nonlinear equation of motion involves the quadratic and cubic terms derived using the Newtonian method and applying the coordinate transformation. The equation of motion is discretized via the Galerkin technique. Results are obtained for the influence of variable structure acceleration and length-scale parameters in the context of the bifurcation theory and on the micro size beam nonlinear characteristics. A nonlinear hardening behavior is observed in the beam’s response. Increasing the magnitude of the applied force tends to signify the nonlinear hardening behavior. At a lower amplitude of the applied force, the transverse response is a one-period stable oscillation. As the amplitude of the excitation force increases, the parameter results in a higher value of the excitation frequency corresponding to the maximum amplitude. At higher size parameters, the response moves from chaotic to period-doubling to the stable one-period response. Non-linearity in the hardening behavior in the frequency response increases with increasing the magnitude of axial acceleration. Although the increase in acceleration tends to increase the hardening behavior, the periodicity of the response does not change with the increasing acceleration. The dynamics response of a small-size beam having constant axial tension attached to a moving structure is considered in this paper. It is recommended to investigate the dynamic response of a small-size beam with variable axial force in the future.

## Figures and Tables

**Table 1:** Nondimensional physical parameters.

Parameter	Definition
$\zeta =\frac{AG{\ell_{s}}^{2}}{EI}$	Small-size parameter
$\alpha =\frac{AE}{P}$	Stretching parameter
$\hat{a}=a$ ρAL^3EI	Nondimensional axial acceleration
$\hat{P}=\frac{PL^{2}}{EI}$	Nondimensional axial force
$T=L^{2}\sqrt{\frac{\rho A}{EI}}$	Nondimensional time parameter
Ω=ωT	Nondimensional frequency

**Table 2:** Values of the simulations’ governing parameters.

Parameter	Values
$\ell_{s}$	$\left(0.0,0.5,1.0\;\right)h$
$\hat{a}$	(0.0, 0.1, 0.3)
F	(0–100) *μN*
Ω	(0–50)

## Data Availability

The data that support the findings of this study are available on request from the corresponding author. he data are not publicly available due to privacy or ethical restrictions.

## References

[1] Wicker J.A., Mote C.D. (1988). Current Research on the Vibration and Stability of Axially-Moving Materials. Shock. Vib. Dig..

[2] Paidoussis M.P. (1998). Fluid-Structure Interactions: Slender Structures and Axial Flow.

[3] Gao H., Yang B. (2020). Dynamic Response of a Beam Structure Excited by Sequentially Moving Rigid Bodies. Int. J. Struct. Stab. Dyn..

[4] Xu C., Wang Z., Li B. (2021). Dynamic Stability of Simply Supported Beams With Multi-Harmonic Parametric Excitation. Int. J. Struct. Stab. Dyn..

[5] Hawwa M.A., Ali S., Hardt D.E. (2019). Influence of Roll-to-Roll System’s Dynamics on Axially Moving Web Vibration. J. Vibroeng..

[6] Ulsoy A.G. (1986). Coupling between Spans in the Vibration of Axially Moving Materials. J. Vib. Acoust. Stress Reliab..

[7] Tang Y.-Q., Ma Z.-G. (2019). Nonlinear Vibration of Axially Moving Beams with Internal Resonance, Speed-Dependent Tension, and Tension-Dependent Speed. Nonlinear Dyn..

[8] Tang Y.-Q., Ma Z.-G., Liu S., Zhang L.-Y. (2019). Parametric Vibration and Numerical Validation of Axially Moving Viscoelastic Beams with Internal Resonance, Time and Spatial Dependent Tension, and Tension Dependent Speed. J. Vib. Acoust..

[9] Chen L.-Q., Yang X.-D. (2005). Steady-State Response of Axially Moving Viscoelastic Beams with Pulsating Speed: Comparison of Two Nonlinear Models. Int. J. Solids Struct..

[10] Ali S., Khan S., Jamal A., Horoub M.M., Iqbal M., Onyelowe K.C. (2021). Transverse Response of an Axially Moving Beam with Intermediate Viscoelastic Support. Math Probl. Eng..

[11] Zhang Z., Yang H., Guo Z., Zhu L., Liu W. (2022). Nonlinear Vibrations of an Axially Moving Beam with Fractional Viscoelastic Damping. Adv. Civ. Eng..

[12] Ali S., Hawwa M.A. (2023). Dynamics of Axially Moving Beams: A Finite Difference Approach. Ain Shams Eng. J..

[13] Bichay N.A., Elaikh T.E. (2021). Transverse Vibration of Cracked Graded Shear Beam with Axial Motion. Int. J. Energy Environ..

[14] Cao T., Hu Y. (2023). Magnetoelastic Primary Resonance and Bifurcation of an Axially Moving Ferromagnetic under Harmonic Magnetic Force. Commun. Nonlinear Sci. Numer. Simul..

[15] Hua H. (2022). Transient Dynamics of an Axially Moving Beam Subject to Continuously Distributed Moving Mass. J. Vib. Eng. Technol..

[16] Öz H.R. (2001). On the Vibrations of an Axially Travelling Beam on Fixed Supports with Variable Velocity. J. Sound Vib..

[17] Öz H.R. (2003). Natural Frequencies of Axially Travelling Tensioned Beams in Contact with a Stationary Mass. J. Sound Vib..

[18] Lim C.W., Li C., Yu J.-L. (2010). Dynamic Behaviour of Axially Moving Nanobeams Based on Nonlocal Elasticity Approach. Acta Mech. Sin..

[19] Li C. (2013). Size-Dependent Thermal Behaviors of Axially Traveling Nanobeams Based on a Strain Gradient Theory. Struct. Eng. Mech. Int. J..

[20] Kiani K. (2014). Longitudinal and Transverse Instabilities of Moving Nanoscale Beam-like Structures Made of Functionally Graded Materials. Compos. Struct..

[21] Sheykhi M., Eskandari A., Ghafari D., Ahmadi Arpanahi R., Mohammadi B., Hosseini Hashemi S. (2023). Investigation of Fluid Viscosity and Density on Vibration of Nano Beam Submerged in Fluid Considering Nonlocal Elasticity Theory. Alex. Eng. J..

[22] Ali S., Hawwa M.A. (2019). A Parametric Study on the Dynamics of Two-Span Roll-to-Roll Microcontact Printing System. Sādhanā.

[23] Ali S., Hawwa M.A., Hardt D.E. (2020). Vibration Suppression of an Axially Moving Web in a Multi-Span Roll-to-Roll Microcontact Printing System. J. Vib. Eng. Technol..

[24] Ali S., Hawwa M.A., Hardt D.E. (2022). Dynamic Behavior of Axially Moving Web in Multi-Span Roll-to-Roll Microcontact Printing System. Proc. Inst. Mech. Engineers. Part I J. Syst. Control Eng..

[25] Zhang D., Tang Y., Chen L. (2019). Nonlinear Vibrations of Axially Moving Beams with Nonhomogeneous Boundary Conditions. Lixue Xuebao Chin. J. Theor. Appl. Mech..

[26] Li C. (2017). Nonlocal Thermo-Electro-Mechanical Coupling Vibrations of Axially Moving Piezoelectric Nanobeams. Mech. Based Des. Struct. Mach..

[27] Marynowski K. (2015). Axially Moving Microscale Panel Model Based on Modified Couple Stress Theory. J. Nanomech. Micromech..

[28] Dehrouyeh-Semnani A.M., Dehrouyeh M., Zafari-Koloukhi H., Ghamami M. (2015). Size-Dependent Frequency and Stability Characteristics of Axially Moving Microbeams Based on Modified Couple Stress Theory. Int. J. Eng. Sci..

[29] Damghanian R., Asemi K., Babaei M. (2022). A New Beam Element for Static, Free and Forced Vibration Responses of Microbeams Resting on Viscoelastic Foundation Based on Modified Couple Stress and Third-Order Beam Theories. Iran. J. Sci. Technol. Trans. Mech. Eng..

[30] Nazari H., Babaei M., Kiarasi F., Asemi K. (2021). Geometrically Nonlinear Dynamic Analysis of Functionally Graded Material Plate Excited by a Moving Load Applying First-Order Shear Deformation Theory via Generalized Differential Quadrature Method. SN Appl. Sci..

[31] Liu J.J., Li C., Fan X.L., Tong L.H. (2017). Transverse Free Vibration and Stability of Axially Moving Nanoplates Based on Nonlocal Elasticity Theory. Appl. Math. Model..

[32] Rezaee M., Lotfan S. (2015). Non-Linear Nonlocal Vibration and Stability Analysis of Axially Moving Nanoscale Beams with Time-Dependent Velocity. Int. J. Mech. Sci..

[33] Liu J., Li C., Yang C., Shen J., Xie F. (2017). Dynamical Responses and Stabilities of Axially Moving Nanoscale Beams with Time-Dependent Velocity Using a Nonlocal Stress Gradient Theory. J. Vib. Control.

[34] Li C., Yu Y.M., Fan X.L., Li S. (2015). Dynamical Characteristics of Axially Accelerating Weak Visco-Elastic Nanoscale Beams Based on a Modified Nonlocal Continuum Theory. J. Vib. Eng. Technol..

[35] Ali S. (2023). Nonlinear Dynamic and Stability of a Small Size Moving Beam under Thermal Conditions. Math. Methods Appl. Sci..

[36] Moaaz O., Abouelregal A.E., Alsharari F. (2023). Lateral Vibration of an Axially Moving Thermoelastic Nanobeam Subjected to an External Transverse Excitation. AIMS Math..

[37] Ji C., Yao L., Li C. (2020). Transverse Vibration and Wave Propagation of Functionally Graded Nanobeams with Axial Motion. J. Vib. Eng. Technol..

[38] Michaltsos G.T. (2010). Bouncing of a Vehicle on an Irregularity: A Mathematical Model. J. Vib. Control.

[39] Shi X.M., Cai C.S. (2009). Simulation of Dynamic Effects of Vehicles on Pavement Using a 3D Interaction Model. J. Transp. Eng..

[40] Lak M.A., Degrande G., Lombaert G. (2011). The Effect of Road Unevenness on the Dynamic Vehicle Response and Ground-Borne Vibrations Due to Road Traffic. Soil Dyn. Earthq. Eng..

[41] Barbosa R.S. (2012). Vehicle Vibration Response Subjected to Longwave Measured Pavement Irregularity. J. Mech. Eng. Autom..

[42] Ghaith F.A. Nonlinear Dynamic Modeling of Elastic Beam Fixed on a Moving Cart and Carrying Lumped Tip Mass Subjected to External Periodic Force. Proceedings of the International Design Engineering Technical Conferences and Computers and Information in Engineering Conference.

[43] Ragulskis K., Gegeckienė L., Kibirkštis E., Miliūnas V., Zubrickaitė L., Pauliukas A., Ragulskis L.M. (2012). Dynamic Study of Transportation Containers with Packages. J. Vibroeng..

[44] Gim G. (1988). Vehicle Dynamic Simulation with a Comprehensive Model for Pneumatic Tires.

[45] Lyasko M.I. (1994). The Determination of Deflection and Contact Characteristics of a Pneumatic Tire on a Rigid Surface. J. Terramech..

[46] An C., Su J. (2014). Dynamic Response of Axially Moving Timoshenko Beams: Integral Transform Solution. Appl. Math. Mech..

